# The Uptake of Screening for Type 2 Diabetes and Prediabetes by Means of Glycated Hemoglobin versus the Oral Glucose Tolerance Test among 18 to 60-Year-Old People of South Asian Origin: A Comparative Study

**DOI:** 10.1371/journal.pone.0136734

**Published:** 2015-08-28

**Authors:** Irene G. M. van Valkengoed, Everlina M. A. Vlaar, Vera Nierkens, Barend J. C. Middelkoop, Karien Stronks

**Affiliations:** 1 Department of Public Health, Academic Medical Centre, University of Amsterdam, Amsterdam, The Netherlands; 2 Department of Public Health, Leiden University Medical Centre, Leiden, The Netherlands; 3 Public Health Service, The Hague, The Netherlands; University of Milan, ITALY

## Abstract

**Background:**

Direct comparisons of the effect of a glycated haemoglobin measurement or an oral glucose tolerance test on the uptake and yield of screening in people of South Asian origin have not been made. We evaluated this in 18 to 60-year-old South Asian Surinamese.

**Materials and Methods:**

We invited 3173 South Asian Surinamese for an oral glucose tolerance test between June 18^th^ 2009- December 31^st^ 2009 and 2012 for a glycated hemoglobin measurement between April 19^th^ 2010-November 11^th^, 2010. Participants were selected from 48 general practices in The Hague, The Netherlands. We used mixed models regression to analyse differences in response and participation between the groups. We described differences in characteristics of participants and calculated the yield as the percentage of all cases identified, if all invitees had been offered screening with the specified method.

**Results:**

The response and participation in the glycated hemoglobin group was higher than in the group offered an oral glucose tolerance test (participation 23.9 vs. 19.3; OR: 1.30, 95%-confidence interval1.01–1.69). After adjustment for age and sex, characteristics of participants were similar for both groups. Overall, glycated hemoglobin identified a similar percentage of type 2 diabetes cases but a higher percentage of prediabetes cases, in the population than the oral glucose tolerance test.

**Conclusion:**

We found that glycated hemoglobin and the oral glucose tolerance test may be equally efficient for identification of type 2 diabetes in populations of South Asian origin. However, for programs aimed at identifying people at high risk of type 2 diabetes (i.e. with prediabetes), the oral glucose tolerance test may be a less efficient choice than glycated hemoglobin.

## Background

Populations of South Asian origin are known to be at particularly high risk of type 2 diabetes mellitus and complications, and are thus an important target for active screening and prevention [1–5]. A recent study has calculated that a substantial benefit can potentially be realised by specifically targeting South Asian populations for active screening and prevention [4].

The potential benefit efficiency/effectiveness of such an approach may in a large part depend on the uptake [6]. Furthermore, studies have shown that the uptake may be influenced by the method used for screening, with less invasive methods associated with a higher uptake [7–12]. The oral glucose tolerance test (OGTT) was for years considered the criterion standard for diagnosis of type 2 diabetes, and several studies have used this method for screening, sometimes after pre-selection based on fasting plasma glucose or risk scores [4, 13–16]. Nevertheless, the concern has been expressed that the decision to commit participants to an arduous OGTT negatively affected the uptake of screening, and that more people would be tested and diagnosed if a more convenient test had been used [4, 6].

In recent years, recommendations have been updated to include glycated hemoglobin (HbA1c) as a diagnostic option [17–19]. Because Hba1c can be determined with a single blood sample, it has practical advantages and is less burdensome than the OGTT, and may therefore be associated with a higher participation in screening and ultimately a higher yield of screening. If that is indeed the case, use of Hba1c could potentially have an important impact on efficiency of screening in the South Asian origin population. However, the possible effect on the uptake and yield of screening has not been evaluated in this high-risk population [4–5, 20].

Therefore, the main aim of our study was to investigate the difference in the uptake, defined by the response to the invitation and participation in the screening, between 18–60 year old South Asian Surinamese men and women offered screening by means of an HbA1c measurement and those offered screening by means of an OGTT. We evaluated whether differences were consistent across age and sex groups. Moreover, we analysed whether a different subset of the population was reached, by analysing whether the characteristics of the screened participants differed according to the screening method. Finally, we estimated the yield of by comparing the percentage of cases of type 2 diabetes mellitus and prediabetes in our population identified if HbA1c as compared to the OGTT had been used.

## Materials and Methods

### Study population

We analysed data on South Asian Surinamese men and women, aged 18–60 years, who were invited to participate in the screening that took place to identify potential participants to be invited for the DH!AAN study, a randomized controlled trial of a lifestyle intervention for the prevention of type 2 diabetes mellitus in the Netherlands (Dutch Trial Register: NTR1499; [21–22]).

The term South Asian Surinamese is used to refer to people with South Asian ancestral origin, and their offspring who migrated to the Netherlands via Suriname. The South Asian Surinamese are the descendants of the indentured labourers from North India—Uttar Pradesh, Uttaranchal and West Bihar- between 1873 and 1917. The two large migration waves, around 1975 and 1980, of South Asian Surinamese to the Netherlands were mainly due to the political situation in Suriname [23].

### Recruitment strategy

We selected 10,420 South Asian Surinamese, aged 18–60 years, from 48 general practice lists in The Hague by means of name analysis. The researcher, the physician or the practice nurse, and a trained research assistant of South Asian Surinamese origin analysed the names. People known to have type 2 diabetes mellitus and pregnant women were excluded.

The general practitioner sent each potential participant an invitation letter with a reply card that could be returned if further contact was unwanted. In DH!AAN, all people invited and screened between May 18^th^, 2009 and April 18^th^ 2010 were offered an OGTT (n = 8408). To account for the possible effect of start-up problems on the recruitment, we excluded those invited before June 18^th^ 2009. We also excluded those invited after December 31^st^ 2009 to reduce the overflow from the OGTT group to the HbA1c measurement period. Thus, the OGTT group consisted of n = 3173 invitees. People invited from April 19^th^ 2010 to November 11^th^, 2010 were offered screening by means of an HbA1c measurement (n = 2012, HbA1c group). We are able to make this comparison between methods due to an abrupt change in the study protocol [21]. Due to the shorter duration of a screening with a single measurement, a greater number of people could be screened within the available time.

The invitations for both groups were similar in content and form, except for the measurement offered. The OGTT group was informed that ‘blood sugar’ would be determined, after which a ‘sugar drink test’ would be performed that consisted of consumption of a ‘sugar drink’ and a ‘measurement of the blood sugars after 2 hours’. Invitees were also informed that, while they waited, their weight, height, waist circumference and blood pressure were measured. The HbA1c group was informed that their weight, height, blood pressure and ‘blood sugar’ would be determined. Screening in both groups took place from Monday to Saturday, after an overnight fast from 10 p.m.

Invitees who had not responded to the invitation within 2 weeks received a written reminder inviting the recipient to make an appointment. The reminder also said that if there was no response (no appointment or reply card), the invitee would be contacted by telephone. The study team phoned those who had not responded within 1 week at least 3 times. If no telephone number was available (24.6% of participants) or potential participants could not be reached, we sent a second written reminder.

All people who made an appointment for screening (‘response’) received a letter of confirmation. In addition, a text message was sent the day before the screening to remind the participants of their appointment.

The materials (e.g. using the colours of the Surinamese flag in the logo of the study, adjusting the risk information in the invitation to the population) and the recruitment strategy used, were based on the approach tested in a previous pilot study [22].

### Data collection screening

People who attended the screening (‘participation’) were requested to fill out a brief questionnaire. We collected data on generation (first/second), education level (primary or lower, secondary, lower vocational, higher vocational or more), paid work (yes/no), known cardiovascular risk (previous diagnosis of high blood pressure, previous diagnosis of high cholesterol or experienced complaints related to heart disease; yes/no), and family history of type 2 diabetes mellitus (yes/no).

Trained research staff carried out a physical examination using a standardized protocol. The participants were weighed in light clothing on a Seca mechanical scale to the nearest 500 g (SECA gmbh & co, Hamburg, Germany). Height was recorded to the nearest 0.01 m on a Seca portable stadiometer. The anthropometric measurements were obtained twice, and the means were used for analysis. From weight and height we calculated the body mass index (BMI; weight in kilograms/height in meters^2^). Blood pressure (Omron M5-1; Omron Healthcare Europe BV, Hoofddorp, the Netherlands) was measured in the seated position around the non-dominant arm supported at heart level. At most, five measurements were taken. We calculated the mean of the first two measurements with less than 5 mm Hg difference in both systolic and diastolic blood pressure [24]. Hypertension was defined as a systolic blood pressure ≥ 140 mm Hg, or a diastolic blood pressure ≥ 90 mm Hg, or a self-reported previous diagnosis of hypertension.

Finally, the participants in both groups were asked to give a fasting blood sample for the measurement of HbA1c and fasting plasma glucose and, in the OGTT group only, to undergo an OGTT (glucose load 75 g). The laboratory methods used have been described previously [25]. Type 2 diabetes mellitus and prediabetes were subsequently classified [17]:

1. according to HbA1c: HbA1c ≥ 48 mmol/mol (≥ 6.5%) as type 2 diabetes, and 39 ≤ HbA1c <48 mmol/mol (5.7 to 6.5%) as prediabetes.
2. according to fasting plasma glucose: a fasting plasma glucose of 126 mg/dl (7.0 mmol/l) or more as type 2 diabetes mellitus and fasting plasma glucose of 100–125 mg/dl (5.6–6.9 mmol/l) as prediabetes.
3. according to the OGTT: type 2 diabetes mellitus by a fasting plasma glucose of 126 mg/dl (7.0 mmol/l) or more and/or 2-h plasma glucose of 200 mg/dl (11.1 mmol/l) or more, and prediabetes (prediabetes) by fasting plasma glucose of 100–125 mg/dl (5.6–6.9 mmol/l) and /or a 2-h glucose post load of 140–199 mg/dl (7.8–11.1 mmol/l).

### Statistical analysis

We calculated the response and participation rates for the HbA1c group and the OGTT group, in the total population and stratified by age and sex. In addition, we described the reasons quoted for non-response. Differences between groups in response, participation and reasons for non-response were assessed with Pearson Chi square tests. We also used logistic regression analysis to calculate the age and sex adjusted odds ratios (with corresponding 95%-confidence intervals) for response and participation in the HbA1c versus the OGTT group. Because we expected variation across general practices in the response [26], we used two-level regression models for these analyses. In these models, participants (level 1) were nested within general practice (level 2). To allow for dependencies between participants registered with the same practice, a random intercept (level 2) was incorporated into the model. In our tables, we report the estimates for the associations derived from these analyses. We also tested for interaction by age or sex by including an interaction term for age* type of invitation and age* type of invitation into the models. A p-value lower than 0.05 for the F test was considered indicative of interaction. Subsequently, we described the differences in the prevalence of characteristics of participants in the HbA1c group and the OGTT group. We tested differences between the groups with Pearson chi-square tests or analysis of variance. We then used two-level regression models to calculate the odds ratio for belonging to the OGTT versus HbA1c group, adjusted for sex and age. We also report the p-values derived from the F-tests for each of the fixed effects.

Finally, we calculated the percentage of new cases of type 2 diabetes mellitus and prediabetes identified per strategy. We estimating the percentage that would have been detected if specified strategy had been used, assuming a similar risk among participants and non-participants. For instance, the percentage of cases detected for the HbA1c strategy was calculated as: the participation rate in HbA1c group times the estimated prevalence defined by HbA1c, divided by the total prevalence as defined by HbA1c and OGTT combined. We defined a range by repeating the calculations with the lower bound and, then, upper boud of the confidence interval estimates for participation and prevalence.

The analyses were performed using the SAS package, version 9.3 (SAS Institute Inc., Cary, USA.). A p-value <0.05 was considered statistically significant. The data underlying our analyses have been made available in (S1 Dataset. Data on response and participation in screening DHIAAN).

### Ethical approval

The Institutional Review Board of the Academic Medical Centre of the University of Amsterdam approved the study. The study was carried out conform the Declaration of Helsinki. All participants provided oral and written informed consent.

## Results

Of those invited, 48,8% were men. The mean age of invitees was 37.3 (37.0–37.6); 29.3% were 18–29 years old, 40.2% 30–44 years and 30.3% 45 years or older.

We observed a slightly higher response and participation rate in the HbA1c group than in the OGTT group (Table 1). The response rate in the HbA1C group was 29.2% (CI 27.6–30.8) versus 24.6% (CI 23.1–26.1) in the OGTT group (p = 0.0002), and the corresponding participation rate 23.9% (CI 22.0–25.8) in the HbA1c group and 19.3% (CI 18.8–21.6) in the OGTT group (p<0.0001). This pattern of differences between the HbA1c and OGTT groups was also observed among men and women and across age groups (Table 1). In both the OGTT group and the HbA1c group, the most frequently cited reason for non-response was ‘no time’ or ‘not interested’, followed by ‘not eligible’ (Table 2). The differences in reasons for non-response between the groups were small and not significant (p = 0.26).

**Table 1:** Differences in response and participation between the HbA1c group and the OGTT group. OGTT group = people offered screening by means of an oral glucose tolerance test; HbA1c group = people offered screening by means of a glycated hemoglobin measurement; Adjusted OR = odds ratio for response or participation, adjusted for age and sex; CI = confidence interval.

		HbA1c group,N = 2012	OGTT group,N = 3173		
		% responders or participants	% responders or participants	Adjusted OR(95%-CI)	P-valueinteract
**Response**		29.2	24.6 ^a^	1.30 (1.00–1.68) ^b^	
**- By sex**	Men	24.7	20.1	1.35 (1.00–1.83)	0.59
	Women	33.7	28.9	1.26 (0.95–1.67)	
**- By agegroup**	18–29 years	20.9	15.2	1.54 (1.09–2.17)	0.05
	30–44 years	28.0	26.8	1.08 (0.80–1.46)	
	45–60 years	39.1	30.7	1.48 (1.08–2.02)	
**Participation**		23.9	19.3 ^a^	1.30 (1.01–1.69) ^b^	
**- By sex**	Men	20.4	15.8	1.36 (1.01–1.84)	0.64
	Women	27.4	22.5	1.27 (0.96–1.68)	
**- By agegroup**	18–29 years	14.8	9.9	1.58 (1.09–2.29)	0.28
	30–44 years	22.8	19.9	1.17 (0.87–1.58)	
	45–60 years	34.4	27.3	1.38 (1.01–1.86)	

^a^ P-value Pearson chi square overall response p = 0.0002 and overall participation p<0.0001;

^b^ P-value F test overall response p = 0.049 and overall participation p = 0.039; P-value interact = p-value from F test of interaction by agegroup or sex; Response = response rate, that is percentage of invitees who made an appointment; participation = participation rate, that is percentage of invitees who were screened; The presented response and participation rates do not account for non-eligibility. The estimated rates accounting for eligibility, by excluding people who were not eligible from the selected population, would be higher.

**Table 2:** Reasons for non-response in the HbA1c group and the OGTT group. OGTT group = people offered screening by means of an oral glucose tolerance test; HbA1c group = people offered screening by means of a glycated hemoglobin measurement; Not eligible = outside age range, longterm illness, currently pregnant, already participating in other research project(s), moved away from The Hague; Unknown = no contact established or no reason provided during telephone contact or on reply card; OGTT = oral glucose tolerance test.

	HbA1c group, N = 1424		OGTT group, N = 2392	
	N	%	N	%
**Not eligible**	255	17.9	370	15.5 ^a^
**Time or interest**	312	21.9	529	22.1
*- No time*, *too busy*	*93*	*6*.*5*	*290*	*12*.*1*
*- No interest or priority*	*219*	*15*.*4*	*239*	*10*.*0*
**Language problems**	3	0.2	6	0.3
**Unknown**	854	60.0	1487	62.2

^a^ P = 0.26 for the Pearson Chi square test for differences in the main categories of reasons for non-response between the OGTT and HbA1c groups.

The age and sex adjusted OR for response in the HbA1c group versus the OGTT group was 1.30 (95%-CI 1.00–1.68). A similar difference between the HbA1c group and the OGTT group was observed for the participation (adjusted OR = 1.30 (95%-CI 1.01–1.69). We did not find evidence for a different association between men and women and between younger and older age groups (Table 1).

Further analysis revealed that the screened population in the HbA1c group was similar to the screened population in OGTT group with regard to age, sex, marital status, level of education, having paid work and known cardiovascular risk, but those in the HbA1c group were less likely to belong to the first generation (p = 0.02) or to have a family history of diabetes than participants in the OGTT group (p = 0.03). After accounting for general practice variation and adjustment for sex and age, the odds of belonging to the HbA1c or OGTT group did not differ significantly between for any of these characteristics (Table 3). The prevalence of overweight, hypertension and the prevalence of type 2 diabetes mellitus and prediabetes defined by fasting plasma glucose or HbA1c were also similar between groups (Table 3).

**Table 3:** Characteristics of participants in the HbA1c group and the OGTT group. OGTT group = people offered screening by means of an OGTT; HbA1c group = people offered screening by means of a glycated hemoglobin measurement; OGTT = oral glucose tolerance test; Adjusted OR = odds ratio for belonging to the OGTT versus HbA1c group, adjusted for age and sex; CI = confidence interval; CVD = cardiovascular disease; T2DM = Type 2 diabetes; Ref = reference group.

		HbA1c group, N = 481	OGTT group, N = 611		
		Mean (sd) or % within group	Mean (sd) or % within group ^a^	Adjusted OR(95%-CI)	P-value
**Age**	in years	41.3 (0.52)	41.4 (0.42)	0.91 (0.14–5.86)	0.94
**Sex**	(men)	42.4	39.6	1.00 (0.92–1.09)	0.92
**Marital status**	(married)	37.6	34.2	0.87 (0.13–5.98)	0.89
**Education**	Primary or less	16.5	14.7	0.84 (0.03–22.90)	0.999
	Secondary	13.6	11.7	0.83 (0.05–12.81)	
	Lower vocational	53.5	56.8	0.76 (0.02–24.79)	
	≥ Higher vocational	16.5	16.8	Ref	
**Paid work**	(yes)	71.5	74.1	0.94 (0.12–7.11)	0.95
**Generation**	(first)	78.3	83.6 ^a^	1.77 (0.09–35.48)	0.71
**Selfreported known CVD risk**	(yes)	37.6	37.2	0.94 (0.13–6.84)	0.95
**Family history T2DM**	(yes)	68.4	74.8 ^a^	1.10 (0.15–8.26)	0.92
**Body mass index**	in kg/m2	26.1 (0.21)	26.1 (0.18)	0.99 (0.81–1.22)	0.95
**Hypertension**	(yes)	40.0	37.0	0.90 (0.13–6.44)	0.92
**Status defined by Hba1c**	T2DM	3.3	3.3 ^b^	1.12 (0.01–172.36)	0.996
	Prediabetes	33.2	37.1	1.09 (0.14–8.52)	
	Normal	63.5	59.6	Ref	
**Status defined by fasting plasma glucose**	T2DM	1.7	2.0 ^b^	1.10 (0.00–855.10)	0.999
	prediabetes	18.5	16.8	0.96 (0.07–13.21)	
	Normal	79.8	81.2	Ref	
**Status defined by OGTT**	T2DM	-	3.5 ^b^	-	-
	prediabetes		21.0		
	Normal		75.5		

^a^ P-values for the univariate differences between groups (Pearson Chi square or ANOVA) are age 0.90, sex 0.35, marital status 0.25, education 0.82, paid work 0.33, generation 0.03, self-reported known CVD risk 0.90, family history T2DM 0.02, body mass index 0.86, hypertension 0.32, Status defined by Hba1c 0.56, Status defined by fasting plasma glucose 0.66;

^b^ Calculated from data on n = 453 people. We excluded 159 people who did not have a full OGTT measurement: n = 13 invitees refused the 2-hour measurement and n = 145 invitees did not have 2-hour measurement because their appointment was scheduled after April 19^th^ 2010, i.e. after the switch to HbA1c as the standard method.

Finally, the percentage of diabetes cases identified in our population appeared to be independent of the strategy used (Table 4). However, the HbA1c strategy did identify more prediabetes cases than the OGTT strategy.

**Table 4:** Estimation of the percentage of the total of cases with type 2 diabetes and prediabetes in the population detected in a 18–60 year old South Asian population in The Hague. Overall prevalence = prevalence based on combined OGTT and HbA1c measurement. OGTT = oral glucose tolerance test; HbA1c = glycated hemoglobin measurement; CI = 95%- confidence interval.

		Type 2 diabetes	Prediabetes
*Overall prevalence* ^a^ ^,^ ^b^		*5*.*1*	*42*.*6*
**If invited for HbA1c**	a) Participation rate (CI)	a) 23.9 (22.0–25.8)	a) 23.9 (22.0–25.8)
	b) Prevalence method (CI) ^b^	b) 3.3 (1.7–4.9)	b) 37.1 (32.8–41.4)
	c) Percentage cases detected (range) ^c^	c) 15.5 (7.4–24.7)	c) 20.8 (17.0–25.1)
**If invited for OGTT**	a) Participation rate (CI)	a) 19.3 (18.8–21.6)	a) 19.3 (18.8–21.6)
	b) Prevalence method (CI) ^b^	b) 3.5 (1.8–5.2)	b) 21.0 (17.2–24.8)
	c) Percentage cases detected (range) ^c^	c) 13.9 (6.7–22.0)	c) 9.9 (7.6–12.6)

^a^Prevalence in total study population, based on diagnosis according to either HbA1c or OGTT,

^b^Calculated from data on n = 453 people with a full OGTT and HbA1c measurement,

^c^Percentage cases detected is calculated as (participation rate x prevalence method)/overall prevalence. To calculate the lower limit of the range we used the lower bound of the confidence interval estimate for participation and prevalence. For the upper limit, we used the upper bounds.

## Discussion

### Main findings

Among men and women and across age groups, we found a higher response and participation among those invited for screening by means of an HbA1c measurement than among those invited for a screening consisting of an OGTT. Although we found that participants in the HbA1c group were less likely to have a family history of diabetes that participants in the OGTT group, no differences were found in other characteristics. The estimated yield, in terms of the percentage of all cases identified if all invitees had been offered screening by means of HbA1c differed from the estimated yield of the OGTT strategy for prediabetes, but not for type 2 diabetes mellitus.

### Discussion of the main findings

#### Uptake of screening

The response and participation rates, regardless of the recruitment strategy, were higher in our study than the rates in two recent studies among South Asian origin populations selected from general practices in the UK [4, 27]. This may be related to the more intensive recruitment strategy used in our study as compared to those studies. As compared to screening studies among European populations, the uptake is only slightly lower in our study [4, 6–12]. This lower uptake was expected as a lower participation is often observed in studies among migrant populations in industrialised countries [4, 28–30].

#### Differences in uptake in HbA1c vs. OGTT group

The higher uptake for HbA1c is in line with the assumption that a more burdensome test is associated with a lower response [4, 20]. It is also in line with the observation from the ADDITION study that participation appeared lower among those offered an OGTT screening than among those offered other methods [7]. However, direct comparisons could not be made in that study as the different methods were used in different populations in different settings. Our findings, however, indicate that the absolute difference in uptake between both methods in this population is relatively small. Interestingly, the burden was not substantially more frequently quoted (reason declined ‘no time or interest’) among non-responders in the OGTT group than in the HbA1c group. Unfortunately, this self-reported reason may not be an adequate reflection of the invitees’ most important considerations for declining participation. Previous studies have demonstrated that participation in preventive programs is influenced by many other factors, such as perceived risk [30–33]. However, perceived risk and other possible factors that nay influence participation in screening were not available for non-participants in our study.

#### Selective participation

One important question is whether the two methods appeal to or reach different subsets of the population, as selection of a subgroup at a higher or lower risk of type 2 diabetes mellitus may affect the efficiency of screening. A relatively higher participation of people with a family history of diabetes in the OGTT group would fit the finding that people with a higher risk perception, related to having a family member with type 2 diabetes, may be more motivated to participate in prevention programmes than others [32–34] However, the differences in the odds of belonging to the HbA1c or OGTT group were not significant after adjustment for age and sex and accounting for general practice variations. Moreover, we did not find differences in the prevalence of type 2 diabetes, prediabetes and other metabolic outcomes between the groups.

#### Yield of screening

Ideally, a screening method efficiently identifies people with previously undiagnosed type 2 diabetes mellitus and, in light of the potential effectiveness of early lifestyle intervention [35], people at risk of type 2 diabetes (e.g. those with prediabetes). Based on the higher uptake, an invitation for screening by means of an Hba1c measurement would seem a better strategy than screening by means of an OGTT. However, the yield for 2 diabetes appeared similar for both strategies. This difference between uptake and yield is likely related to the estimated prevalence of type 2 diabetes, and the overlap of measures in our population. We have previously shown that Hba1c may not detect all new cases in our population that would have been identified if the OGTT had been used and vice versa [25].

While the estimated yield was similar for type 2 diabetes, the yield did differ between strategies for prediabetes. This difference between outcomes may be related to the greater correlation between both measures among people with diabetes than in those without diabetes [36]. In particular, because we chose the American Diabetes Association criteria to classify ‘prediabetes’, which use a broader range to define impaired glucose regulation as compared to other criteria [17, 18]. The broader criteria result in a markedly higher proportion of people only identified with HbA1c as having prediabetes [37]. This is proportionally reflected in our calculation of the yield. However, as the uptake and prevalence in other populations or settings may be different, replication of our findings is warranted before a final conclusion is drawn.

### Limitations

We made a comparison between potential invitees who were invited for a screening including an OGTT and people who were not. This ‘natural experiment’ was the result of an abrupt change in the study protocol [21]. However, a non-randomised comparison has some potential drawbacks. For instance, the difference in participation in the two groups may have been affected by changes in external circumstances. However, the recruitment took place in a relatively short period during which there were no major changes in local circumstances, such as local screening policies. Thus, it is unlikely that this explains the differences between the groups.

Another potential problem may be that the results were affected by baseline differences in characteristics that we did not measure. In addition, the uptake may be affected by characteristics of the general practices that participants were registered with [26]. We attempted to account for this variation by using multilevel analyses.

In our study, we asked all potential participants to fast prior to the screening appointment. However, fasting is not required for an HbA1c measurement [17–19]. The difference in uptake with the OGTT group might have been larger if we had dropped the requirement to fast for the HbA1c group.

Finally, we assumed a similar prevalence among people who were not screened than among people screened in our calculations of the yield, while this may not be the case [38]. This may have affected the absolute estimates of the yield if people with or without disease were more or less inclined to respond to an invitation for an OGTT measurement than for an HbA1c measurement. However, the comparison of the characteristics of screened participants did not show differences in metabolic profile between groups. Therefore, we expect that the effect on our results is small.

## Conclusions

An invitation for screening with HbA1c was associated with a slightly higher uptake of screening in population of South Asian origin than an invitation for an OGTT, but the methods did not appeal to a substantially different subset of the population. The difference between strategies in the yield of screening for prediabetes suggests that the OGTT may be a less efficient than HbA1c for programs aimed at the identification of people at risk of diabetes. For type 2 diabetes, the yield was similar for HbA1c and the OGTT. This suggests that either method may be chosen for type 2 diabetes screening. However, although the yield is an important consideration, the final choice in practice should also be determined by factors that we did not record in our study, such as cost, organisational aspects and patients’ experience.

## Supporting Information

S1 Dataset Data on response and participation in screening DHIAAN. A SAS 9.3 dataset with the data underlying this manuscript.
(SAS7BDAT)

## References

[1] Bindraban NR, van Valkengoed IG, Mairuhu G, Koster RW, Holleman F, Hoekstra JBL et al Prevalence of diabetes mellitus and the performance of a risk score among Hindustani Surinamese, African Surinamese and ethnic Dutch: a cross-sectional population-based study. BMC Public Health 2008;8: 271 10.1186/1471-2458-8-271 18673544 PMC2533321

[2] Mangalmurti SS, Paley A, Gany F, Fisher EA, Hochman JS. South Asians and risk of cardiovascular disease: current insights and trends. Ethn Dis 2010;20: 474–478. 21305840

[3] Gholap N, Davies M, Patel K, Sattar N, Khunti K. Type 2 diabetes and cardiovascular disease in South Asians. Prim Care Diabetes 2011;5: 45–56. 10.1016/j.pcd.2010.08.002 20869934

[4] Webb DR, Gray LJ, Khunti K, Srinivasan B, Taub N, Campbell S, et al Screening for diabetes using an oral glucose tolerance test within a Western multi-ethnic population identifies modifiable cardiovascular risk: the ADDITION-Leicester study. Diabetologia 2011;54: 2237–46. 10.1007/s00125-011-2189-2 21638133

[5] Stronks KS, van Valkengoed IGM. How can we realise the potentially large public health benefit of screening for type 2 diabetes mellitus in south Asians? Diabetologia 2011; \54: 2214–2216. 10.1007/s00125-011-2258-6 21769508 PMC3149662

[6] Waugh N, Scotland G, McNamee P, Gillett M, Brennan A, Goyder E, et al Screening for type 2 diabetes: literature review and economic modelling. Health Technol Assess 2007;11: iii–iv, ix–xi, 1–125. 10.3310/hta11170 17462167

[7] Van den Donk M, Sandbaek A, Borch-Johnsen K, Lauritzen, Simmons RK et al Screening for type 2 diabetes. Lessons from the ADDITION-Europe study. Diabet Med. 2011;28: 1416–24. 10.1111/j.1464-5491.2011.03365.x 21679235

[8] Lawrence JM, Bennett P, Young A, Robinson AM. Screening for diabetes in general practice: cross sectional population study. BMJ 2001;323: 548–51. 11546702 10.1136/bmj.323.7312.548 PMC48161

[9] Greaves CJ, Stead JW, Hattersley AT, Ewings P, Brown P, Evans PH. A simple pragmatic system for detecting new cases of type 2 diabetes and impaired fasting glycaemia in primary care. Fam Pract. 2004;21: 57–62. 14760046 10.1093/fampra/cmh113

[10] Christensen JO, Sandbaek A, Lauritzen T, Borch-Johnsen K. Population-based stepwise screening for unrecognised Type 2 diabetes is ineffective in general practice despite reliable algorithms. Diabetologia 2004;47: 1566–73. 15365615 10.1007/s00125-004-1496-2

[11] O’Connor PJ, Rush WA, Cherney LM, Pronk NP. Screening for diabetes mellitus in high-risk patients: cost, yield, and acceptability. Eff Clin Pract. 2001;4: 271–7. 11769300

[12] Spijkerman AM, Adriaanse MC, Dekker JM, Nijpels G, Stehouwer CD, Bouter LM, et al Diabetic patients detected by population-based stepwise screening already have a diabetic cardiovascular risk profile. Diabetes Care 2002;25: 1784–9. 12351478 10.2337/diacare.25.10.1784

[13] Dunstan DW, Zimmet PZ, Welborn TA, De Courten MP, Cameron AJ, Sicree RA, et al The rising prevalence of diabetes and impaired glucose tolerance: the Australian Diabetes, Obesity and Lifestyle Study. Diabetes Care 2002;25: 829–34. 11978676 10.2337/diacare.25.5.829

[14] Franciosi M, De Berardis G, Rossi MC, Sacco M, Belfiglio M, Pellegrini F, et al Use of the diabetes risk score for opportunistic screening of undiagnosed diabetes and impaired glucose tolerance: the IGLOO (Impaired Glucose Tolerance and Long-Term Outcomes Observational) study. Diabetes Care 2005;28: 1187–94. 15855587 10.2337/diacare.28.5.1187

[15] Davies MJ, Ammari F, Sherriff C, Burden ML, Gujral J, Burden AC. Screening for Type 2 diabetes mellitus in the UK Indo-Asian population. Diabet Med 1999;16: 131–7. 10229306 10.1046/j.1464-5491.1999.00012.x

[16] Gray LJ, Khunti K, Williams S, Goldby S, Troughton J, Yates T, et al Let's prevent diabetes: study protocol for a cluster randomised controlled trial of an educational intervention in a multi-ethnic UK population with screen detected impaired glucose regulation. Cardiovasc Diabetol 2012; 11:56 22607160 10.1186/1475-2840-11-56 PMC3431251

[17] American Diabetes Association. Diagnosis and classification of diabetes mellitus. Diabetes Care 2013; 36 Suppl 1: S67–S74 10.2337/dc13-S067 23264425 PMC3537273

[18] International Expert Committee. International Expert Committee report on the role of the A1C assay in the diagnosis of diabetes. Diabetes Care 2009;32: 1327–34. 10.2337/dc09-9033 19502545 PMC2699715

[19] National Institute for Health and Clinical Excellence. Preventing type 2 diabetes: risk identification and interventions for individuals at high risk. NICE [Internet]. 2012 [cited 29 june 2013]. Available: www.nice.org.uk/nicemedia/live/13791/59951/59951.pdf.

[20] Malkani S, Mordes JP. Implications of using hemoglobin A1C for diagnosing diabetes mellitus. Am J Med. 2011;124: 395–401. 10.1016/j.amjmed.2010.11.025 21531226 PMC3086708

[21] Vlaar EM, van Valkengoed IG, Nierkens V, Nicolaou M, Middelkoop BJ, Stronks K. Feasibility and effectiveness of a targeted diabetes prevention program for 18 to 60-year-old South Asian migrants: design and methods of the DH!AAN study. BMC Public Health 2012; 12:371 10.1186/1471-2458-12-371 22621376 PMC3504520

[22] Nicolaou M, Vlaar E, Van Valkengoed I, Middelkoop B, Stronks K, Nierkens V. Development of a diabetes prevention program for Surinamese South Asians in the Netherlands. Health Promot Int, 2014;29: 680–91. 10.1093/heapro/dat018 23564419

[23] Choenni C. Integratie Hindostani Stijl? Over migratie, geschiedenis en diaspora van Hindostanen. Vrije University [Internet]. 2011 [cited 16 february 2013]. Available from: http://hdl.handle.net/1871/19540.

[24] The Working Group on Revised Hypertension Guidelines. Techniques of office blood pressure measurement Revised Hypertension Guidelines. The Dutch Institute for Healthcare Improvement and the Dutch Heart Foundation The Netherlands, 2000.

[25] Vlaar EMA, Admiraal WM, Busschers WB, Holleman F, Nierkens V, Middelkoop BJC, et al Screening South Asians for type 2 diabetes and prediabetes: (1) comparing oral glucose tolerance and haemoglobin A1c test results and (2) comparing the two sets of metabolic profiles of individuals diagnosed with these two tests. BMC Endocr Disord. 2013;13: 8 10.1186/1472-6823-13-8 23442875 PMC3700889

[26] Sargeant LA, Simmons RK, Barling RS, Butler R, Williams KM, Prevost AT, et al Who attends a UK diabetes screening programme? Findings from the ADDITION-Cambridge study. Diabet Med. 2010;27: 995–1003. 10.1111/j.1464-5491.2010.03056.x 20722672 PMC3428846

[27] Douglas A, Bhopal RS, Bhopal R, Forbes JF, Gill JM, Lawton J et al Recruiting South Asians to a lifestyle intervention trial: experiences and lessons from PODOSA (Prevention of Diabetes & Obesity in South Asians). Trials 2011;12: 220 10.1186/1745-6215-12-220 21978409 PMC3201899

[28] Mason S, Hussain-Gambles M, Leese B, Atkin K, Brown J. Representation of South Asian people in randomised clinical trials: analysis of trials' data. BMJ. 2003; 326:1244–1245 12791739 10.1136/bmj.326.7401.1244 PMC161554

[29] Bartlett C, Doyal L, Ebrahim S, Davey P, Bachmann M, Egger M, et al The causes and effects of socio-demographic exclusions from clinical trials. Health Technol Assess. 2005; 9: iii–x. 10.3310/hta9380 16181564

[30] El Fakiri F, Bruijnzeels MA, Foets MM, Hoes AW. Different distribution of cardiovascular risk factors according to ethnicity: a study in a high risk population. J Immigr Minor Health. 2008;10: 559–565. 10.1007/s10903-008-9144-4 18483765

[31] Banerjee AT, Grace SL, Thomas SG, Faulkner G. Cultural factors facilitating cardiac rehabilitation participation among Canadian South Asians: a qualitative study. Heart Lung 2010;39: 494–503. 10.1016/j.hrtlng.2009.10.021 20561846

[32] Gatewood JG, Litchfield RE, Ryan SJ, Geadelmann JD, Pendergast JF, Ullom KK. Perceived barriers to community-based health promotion program participation. Am J Health Behav. 2008;32: 260–71. 18067466 10.5555/ajhb.2008.32.3.260

[33] Toft UN, Kristoffersen LH, Aadahl M, von Huth Smith L, Pisinger C, Jørgensen T. Diet and exercise intervention in a general population—mediators of participation and adherence: the Inter99 study. Eur J Public Health. 2007;17: 455–63. 17170019 10.1093/eurpub/ckl262

[34] Dorman JS, Valdez R, Liu T, Wang C, Rubinstein WS, O'Neill SM, et al Health beliefs among individuals at increased familial risk for type 2 diabetes: Implications for prevention. Diabetes Res Clin Pract. 2012;96: 156–62. 10.1016/j.diabres.2011.12.017 22257420 PMC3905745

[35] Schellenberg ES, Dryden DM, Vandermeer B, Ha C, Korownyk C. Lifestyle interventions for patients with and at risk for type 2 diabetes: a systematic review and meta-analysis. Ann Intern Med. 2013;159: 543–51. 10.7326/0003-4819-159-8-201310150-00007 24126648

[36] van 't Riet E, Alssema M, Rijkelijkhuizen JM, Kostense PJ, Nijpels G, Dekker JM. Relationship between A1C and glucose levels in the general Dutch population: the new Hoorn study. Diabetes Care. 2010;33: 61–6. 10.2337/dc09-0677 19808928 PMC2797987

[37] Mostafa SA, Khunti K, Srinivasan BT, Webb D, Gray LJ, Davies MJ. The potential impact and optimal cut-points of using glycated haemoglobin, HbA1c, to detect people with impaired glucose regulation in a UK multi-ethnic cohort. Diabetes Res Clin Pract. 2010;90: 100–8. 10.1016/j.diabres.2010.06.008 20633944

[38] Coutinho M, Gerstein HC, Wang Y, Yusuf S. The relationship between glucose and incident cardiovascular events. A metaregression analysis of published data from 20 studies of 95,783 individuals followed for 12.4 years. Diabetes Care 1999;22: 233–24. 10333939 10.2337/diacare.22.2.233

